# Durable Scar Size Reduction Due to Allogeneic Mesenchymal Stem Cell Therapy Regulates Whole‐Chamber Remodeling

**DOI:** 10.1161/JAHA.113.000140

**Published:** 2013-06-21

**Authors:** Adam R. Williams, Viky Y. Suncion, Frederic McCall, Danny Guerra, Jacques Mather, Juan P. Zambrano, Alan W. Heldman, Joshua M. Hare

**Affiliations:** 1 Interdisciplinary Stem Cell Institute, University of Miami Miller School of Medicine, Miami, FL (A.R.W., V.Y.S., F.M.C., J.M., J.P.Z., A.W.H., J.M.H.); 2 Department of Medicine, Cardiovascular Division, University of Miami Miller School of Medicine, Miami, FL (D.G., J.P.Z., A.W.H., J.M.H.); 3 Department of Surgery, University of Miami Miller School of Medicine, Miami, FL (A.R.W., J.M.)

**Keywords:** cardiac magnetic resonance imaging, ischemic cardiomyopathy, stem cell therapy

## Abstract

**Background:**

Intramyocardial injection of mesenchymal stem cells (MSCs) in chronic ischemic cardiomyopathy is associated with reverse remodeling in experimental models and humans. Here, we tested the hypothesis that allogeneic MSC therapy drives ventricular remodeling by producing durable and progressive scar size reduction in ischemic cardiomyopathy.

**Methods and Results:**

Gottingen swine (n=12) underwent left anterior descending coronary artery myocardial infarction (MI), and 3 months post‐MI animals received either intramyocardial allogeneic MSC injection (200 mol/L cells; n=6) or left ventricle (LV) catheterization without injection (n=6). Swine were followed with serial cardiac magnetic resonance imaging for 9 months to assess structural and functional changes of the LV. Intramyocardial injection was performed using an integrated imaging platform combining electroanatomical mapping unipolar voltage and 3‐dimensional cardiac magnetic resonance imaging angiography–derived anatomy to accurately target infarct border zone injections. MSC‐treated animals had a 19.62±2.86% reduction in scar size at 3 months postinjection, which progressed to 28.09±2.31% from 3 to 6 months postinjection (*P*<0.0001). MSC‐treated animals had unchanged end‐diastolic volume (EDV;* P*=0.08) and end‐systolic volume (ESV;* P*=0.28) from preinjection to 6 months postinjection, whereas controls had progressive dilatation in both EDV (*P*=0.0002) and ESV (*P*=0.0002). In addition, MSC‐treated animals had improved LV sphericity index. Percentage change in infarct size correlated with percentage change in EDV (*r*=0.68; *P*=0.01) and ESV (*r*=0.77; *P*=0.001). Ejection fraction increased from 29.69±1.68% to 35.85±2.74% at 3 months post‐MSC injection and progressed to 39.02±2.42% 6 months postinjection (*P*=0.0001), whereas controls had a persistently depressed ejection fraction during follow‐up (*P*=0.33).

**Conclusion:**

Intramyocardial injection of allogeneic MSCs leads to a sustained and progressive reduction in infarct size, which in turn drives reverse remodeling and increases in ejection fraction. These findings support ongoing biological activity of cell therapy for substantial periods and suggest optimal end points for future clinical trials.

## Introduction

Myocardial infarction (MI) leads to loss of cardiomyocytes, which are replaced with fibrous scar tissue.^1^ Ischemic cardiomyopathy (ICM), the most common etiology of heart failure, commonly manifests after left anterior descending (LAD) coronary artery MI.^2^ The left ventricle (LV) adapts to the lost myocardium by undergoing significant dilatation to maintain stroke volume and cardiac output.^3^ Increased LV chamber volume and altered chamber configuration from an elliptical to a spherical shape are the hallmarks of ventricular remodeling.^4^ The long‐term prognosis is poor, with progressive enlargement of the heart, and increased LV chamber dimensions are associated with future adverse cardiovascular events and decreased survival.^5–6^ Limited therapies exist to prevent or reverse the progressive dilatation of the LV chamber after MI.

Cell‐based therapy is emerging as a potentially transformative treatment for advanced heart failure. Many cell types isolated from either a bone marrow or cardiac biopsy are currently under investigation in clinical trials.^7–9^ Mesenchymal stem cells (MSCs) were first isolated in the 1970s and are a highly attractive cell therapy for heart failure. MSCs are easily isolated from a bone marrow biopsy, have profound effects on other cells, such as immunomodulation and regulation of cardiac stem cell niches, and can even induce differentiation of other stem cells.^10^ Large animal and early clinical data in subjects with ischemic cardiomyopathy support the ability of MSCs to attenuate and even induce reverse remodeling.^7,11–12^ Although enthusiasm for cell‐based therapy is increasingly supported, very few studies have addressed long‐term effects and sustainability of cell therapy. Importantly, some studies, albeit in patients treated at the time of MI, have suggested that cell‐based therapy may not produce durable effects, with regression of favorable effects over time. With regard to ischemic cardiomyopathy, follow‐up periods for preclinical models are typically in months, whereas in clinical investigations patient follow‐up has not exceeded 1 year. To address this issue, we assessed longer‐term follow‐up in a well‐established porcine model of ischemic cardiomyopathy and sought predictors of long‐term benefit. Here we show that reverse remodeling is not only sustained but actually progressive.

Advances in cardiac magnetic resonance imaging (CMR) have provided unprecedented imaging capabilities to investigate structural changes in the left ventricle (LV) and is considered the “gold standard” for measuring chamber volumes.^4^ CMR allows for highly accurate and detailed cardiac phenotyping in large animal translational studies, but short follow‐up in these studies has limited our understanding of long‐term LV structural changes after MSC therapy. Using merged CMR and electroanatomical 3‐dimensional imaging to guide intramyocadial injection catheters, we tested the hypothesis that allogeneic MSC therapy produces reverse remodeling through durable scar size reduction in ischemic cardiomyopathy.

## Methods

Female Gottingen minipigs (n=12; 23 to 28 kg) underwent closed‐chest ischemia–reperfusion to generate a model of ischemic cardiomyopathy as previously described.^13^ At 3 months post‐MI animals were randomized to injection of 200 million allogeneic MSCs or no injection control (LV catheterization without injection). Serial CMR was performed to assess structural changes in the LV over a 9‐month period. All animal protocols were reviewed and approved by the University of Miami Institutional Animal Care and Use Committee.

### Creation of Myocardial Infarction

Each pig was placed under general anesthesia using ketamine (33 mg/kg intramuscularly) for induction and isoflurane (2% to 3% inhalation) via face mask. Endotracheal intubation was done, and the pig continued on isoflurane (1% to 2% inhalation). Cut‐down of a carotid or femoral artery and vein was done for placement of vascular introducers. Heparin (100 IU/kg) and lidocaine (3 mg/kg) were given intravenously followed by infusion of lidocaine (0.01 mg/kg per minute) for the duration of the infarction.

Using angioplasty techniques, the LAD coronary artery was occluded immediately beyond the first diagonal branch for 150 minutes. After 150 minutes, the angioplasty balloon was deflated and coronary angiography performed to confirm reperfusion. The animal was recovered from anesthesia, and a chronic ischemic cardiomyopathy model developed over the 3 months following infarction.

### Cardiac Magnetic Resonance Imaging

CMR studies were conducted on a Siemens Symphony 1.5T (Erlangen, Germany) scanner with Syngo MR A30 software using a 4‐channel body coil with ECG gating and short breath‐hold acquisitions. Postprocess image analysis was performed with custom QMass (Medis, Leiden, Netherlands) and Segment Software (Medviso AB; Lund, Sweden). All animals underwent CMR at baseline (48 to 72 hours before experimentally induced MI), 3 months post‐MI (stem cell injection time), 6 months post‐MI, and 9 months post‐MI (before euthanizing). Pigs were placed under general endotracheal anesthesia as described above during all CMR procedures.

Steady‐state free precession (SSFP) cine images in 2‐chamber short ‐axis planes (slice thickness, 4 mm; field of view [FOV], 240 to 340 mm; matrix 256×80; repetition time [TR], 52 ms; echo time [TE], 1 ms; number of averages, 4; band width [BW], 900 kHz; flip angle, 64°) were obtained. At end‐diastole and end‐systole, semiautomated epicardial and endocardial borders were drawn in contiguous short‐axis cine images covering the apex to mitral valve plane to calculate end‐diastolic volume (EDV) and end‐systolic volume (ESV), stroke volume (SV), and ejection fraction (EF). Sphericity index (SI) was calculated as the ratio of the LV diastolic volume and systolic volume to the volume of a sphere with the diameter of the long axis of the LV in diastole and systole obtained from a 4‐chamber SSFP image (LV volume/[LV long axis length^3^×π/6]), providing a diastolic and systolic SI value.^14–15^ Three‐dimensional reconstruction sequences were acquired during the first‐pass perfusion phase following intravenous infusion of gadolinium 0.3 mmol/kg (Magnevist, Bayer Healthcare, Wayne, NJ). A series of axial images (≈75 to 100 frames; slice thickness, 1.5 mm with no gap; FOV, 240 to 340 mm; matrix 256×80; TR/TE, 4/1 ms; BW, 360 kHz; and flip angle, 25°) were acquired to create a 3‐D stack of images.

Short‐axis and 2‐chamber long‐axis delayed enhancement (DE) images (slice thickness, 4 mm with no gap; FOV, 240 to 340 mm; matrix 256×80; TR/TE, 450/3 ms; BW, 150 kHz; and flip angle, 25°) were acquired 8 minutes following intravenous infusion of gadolinium 0.3 mmol/kg. Infarct scar size was calculated from the short‐axis delayed myocardial enhancement images covering the apex to the mitral valve plane.^16^ Epicardial and endocardial contours of the LV were drawn with a semiautomated tool. The intensity of a normal region of myocardium was calculated, and scar tissue was determined using an intensity threshold 2 standard deviations above normal myocardium. Delayed‐enhancement CMR images were segmented using a 17‐segment model of the LV. Regions with <50% transmural hyperenhancement were identified as subendocardial scar and segments with >50% hyperenhancement were defined as transmural scar. Segments with no hyperenhancement were identified as normal myocardium. Electroanatomical mapping (EAM) unipolar voltages values in each of 17 segments was correlated with the CMR DE findings, allowing the description of unipolar voltage values in normal myocardium, subendocardial scar, and transmural scar.

### Bone Marrow Mesenchymal Stem Cells

A bone marrow (BM) aspirate was obtained from the iliac crest of a male swine. MSCs were isolated from other BM cells by Ficoll density centrifugation and plastic adherence as previously described.^17^ MSCs were amplified, harvested, and cryopreserved. On the morning of stem cell injection, cells were thawed, washed, and resuspended in 5 mL of phosphate buffered saline.

### CMR‐EAM Intramyocardial Injection of Allogeneic MSCs

EAM uses a magnetic field that guides an intramyocardial catheter that measures magnetic field strength in the *x*,* y*, and *z* planes, thus capable of creating a 3‐dimensional reconstruction of the heart.^18^ In addition, a local electrocardiogram can be obtained to provide real‐time myocardial voltage data to guide catheters to damaged areas of the LV. EAM was done in an angiography suite using a NOGA XP Cardiac Navigation System (Biologics Delivery Systems Group, Irwindale, CA) using custom merge software. A NOGASTAR mapping catheter acquired local electrocardiograms, and a MYOSTAR catheter was used for intramyocardial injection of stem cells.

Animals were placed under general anesthesia as previously described. Cut‐down of the carotid or femoral vasculature was done for placement of 8 French and 9 French vascular introducers into the artery and vein, respectively. EAM was acquired via a catheter inserted retrograde into the LV under fluoroscopic guidance. The mapping catheter tip was directed to acquire a series of endocardial contact points, and a 3‐dimensional map of the LV was constructed showing corresponding unipolar electrocardiographic voltage. Points (typically 90 to 120) encompassing the anterior, septal, inferior, and lateral walls were acquired to create the map.

The gadolinium‐enhanced axial CMR angiography images were transferred to a EAM workstation. The heart and great vessels were reconstructed and LV segmented for image integration with the EAM to exclude other structures. Manual landmark and automated surface registration were used to integrate the CMR and EAM images; Automated surface registration was done using endocardial surface contour alignment. Image integration created a merged image of EAM unipolar voltage data projected onto a CMR 3‐dimensional reconstruction of the LV.

Once the CMR‐EAM merged image was created, the injection catheter was guided by fluoroscopy into the LV. The target area of the injections was the infarct border zone (rim of tissue immediately adjacent to the infarct territory), where 200 million allogeneic bone marrow–derived MSCs were injected into 15 sites. Each injection consisted of a 0.5‐mL aliquot of cells.

Criteria used to confirm engagement of the needle tip within the myocardium were: (1) loop stability <2.5 mm; (2) EAM confirmation of perpendicular placement of catheter tip; and (3) cine fluoroscopy confirmation of catheter stability and perpendicular placement of catheter tip.

### Statistical Analysis

All data are presented as mean (±standard error of mean). Repeated‐measures (RM) ANOVA was used to test differences between means with time postinjection used as the repeated factor, and a Bonferroni correction was used for multiple testing. A 1‐way RM ANOVA was used for within‐group analyses and a 2‐way RM ANOVA for between‐group analyses. GraphPad Prism (Version 4.03, La Jolla, CA) was used to analyze all data points and plot graphs. *P*<0.05 was considered statistically significant.

## Results

### Ventricular Remodeling After Myocardial Infarction

**Figure 1.**
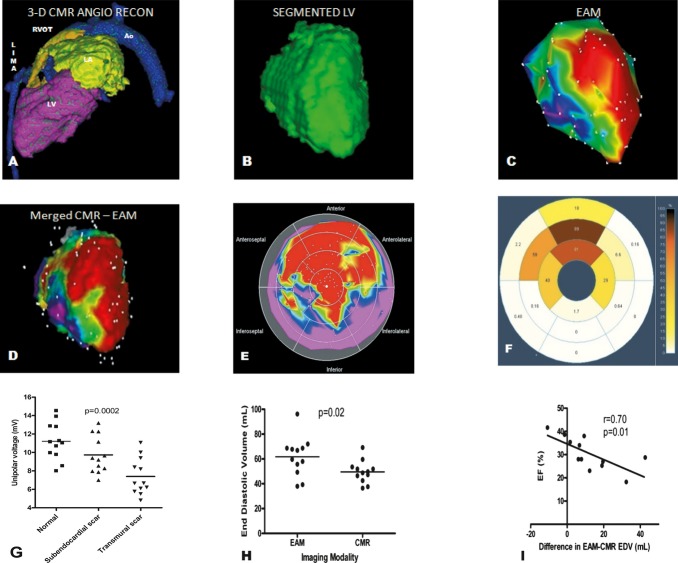
Merging CMR angiography and electroanatomical mapping to guide transendocardial mesenchymal stem cell injection. A, CMR angiography–derived 3‐dimensional reconstruction of the heart and great vessels. B, Segmented CMR LV reconstruction with corresponding (C) EAM unipolar violated LV map. D, Merged CMR‐EAM image showing EAM unipolar voltage projected onto a detailed CMR angiography reconstruction of the LV. Bull's‐eye 17‐segment plot of corresponding (E) EAM unipolar voltage–derived infarct (red indicates scar with depressed voltage) and (F) delayed‐enhancement CMR‐derived scar (darker areas indicate infarct). G, EAM unipolar voltages for scar tissue compared with normal myocardium. H, EAM creates larger LV volumes compared with CMR, which (I) correlated with the degree of LV dysfunction. CMR indicates cardiac magnetic resonance imaging; LIMA, left internal mammary artery; RVOT, right ventricle outflow tract; LV, left ventricle; Ao, aorta; EAM, electroanatomic map; EF, ejection fraction; EDV, end‐diastolic volume; LA, left atrium.

Extensive ventricular remodeling occurred following proximal LAD occlusion–reperfusion injury. EDV increased from 32.8±2.2 to 49.1±2.6 mL, and ESV increased from 16.8±1.1 to 33.7±1.9 mL 3 months post‐MI (n=12; *P*<0.0001). A compensatory parallel increase in EDV with ESV (*r*^2^=0.98, *P*=0.01) occurred to preserve SV (16.0±1.2 versus 15.4±1.4 mL; *P*=0.28), but resulted in significantly depressed EF (48.7±1.3% to 31.2±2.1%; *P*<0.0001). DE‐CMR showed anterior wall scar encompassing 18.27±1.0% of LV mass 3 months post‐MI. Correlation of CMR DE imaging with EAM showed lower unipolar voltage in scarred myocardium (measuring <8 mV for transmural scar and 9 to 10 mV for subendocardial scar) compared with normal myocardium (values >11 mV) (Figure 1).

### CMR‐EAM Stem Cell Injections

All animals that underwent stem cell injection (n=6) and the no‐injection controls (n=6) were alive at 9 months. One swine developed bradycardia and hypotension requiring atropine during MSC injection, and 1 animal had sustained ventricular tachycardia requiring cardioversion. Both pigs completed the stem cell injection procedure and the study protocol without further arrhythmias or adverse events. Integration of CMR with EAM resulted in an average reconstruction difference of 3.8±2.8 mm, with larger EAM‐derived chamber volume compared with CMR angiography (*P*=0.02; Figure 1H). EAM obtains points by placing the catheter tip against the endocardial wall and likely deforms it outward, thus acquiring spatial points that reconstruct a larger LV chamber. Animals with severely depressed EFs had larger differences in volumes, indicating weak and easily deformable scarred chamber walls (Figure 1). Increasing the number of landmark registration points from 2 (apex and anterior wall) to 3 to 4 (apex, anterior wall, and 2 points on the mitral valve annulus) produced similar 3‐D reconstructions (4.3±0.5 versus 4.0±0.5 mm, respectively).

### LV Structural and Functional Changes After MSC Therapy

**Figure 2.**
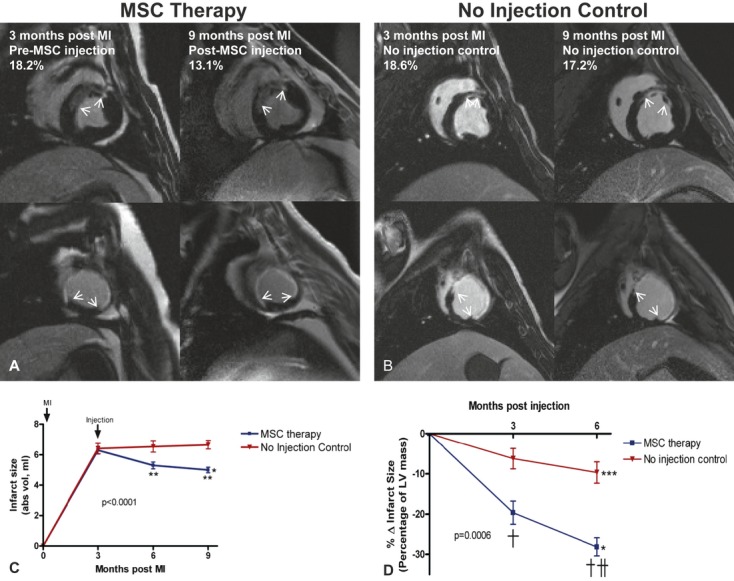
Durable and progressive scar size reduction due to intramyocardial injection of allogeneic bone marrow MSCs. A, Delayed‐enhancement CMR scar size images show durable reduction in scar size with MSC therapy. B, Control animals have stable infarct size in ischemic cardiomyopathy. C, Infarct size was reduced with MSC therapy compared with control when measured by absolute scar volume. D, Scar size reduction with MSC therapy was durable and progressive from 3 to 6 months postinjection as measured by infarct size as a percentage of LV mass. (*P<0.0001,** *P*<0.001 vs 3 months, ***P<0.05, †*P*<0.01 MSC vs placebo, ††*P*<0.05 at 3 vs 6 months; between‐group ANOVA interaction *P* value is plotted in each graph). MSCs indicates mesenchymal stem cells; MI, myocardial infarction; LV, left ventricle; CMR, cardiac magnetic resonance imaging; ANOVA, analysis of variance.

MSC therapy reduced scar size as measured by absolute volume 3 months postinjection and persisted 6 months postinjection (*P*<0.0001; Figure 2C). In MSC‐treated animals, scar size as a percentage of LV mass decreased 19.62±2.86% at 3 months postinjection and progressed to 28.09±2.31% from 3 to 6 months postinjection (*P*<0.0001; Figure 2D). No injection control animals had stable infarct size measured by absolute volume from 3 to 9 months post‐MI (*P*=0.24), whereas scar size as a percentage of LV mass decreased 9.68±2.65% (*P*=0.02) (Figure 2C and 2D).

**Figure 3.**
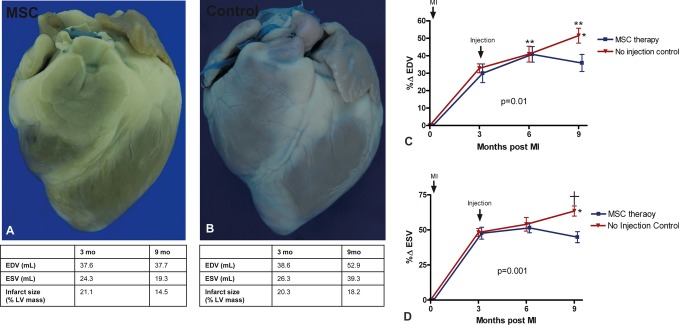
Allogeneic MSC therapy reverses remodeling in ischemic cardiomyopathy. Example of a swine heart (A) 6 months postinjection of allogeneic MSCs compared with a (B) no‐injection control (note: both hearts had comparable EDV, ESV, and infarct size 3 months post‐MI). The control heart demonstrated progressive LV dilatation, whereas the MSC‐treated heart had significantly smaller LV chamber volumes accompanied by reduced infarct size. Graphs showing percentage change in (C) EDV and (D) ESV. No injection control animals had progressive dilatation of both EDV and ESV from 3 months (preinjection point) to 9 months, whereas the MSC‐treated group had stable LV volumes. (*P<0.001, **P<0.05 at 3 vs 6 months and 6 vs 9 months, †*P*<0.01 at 6 vs 9 months; between‐group ANOVA interaction *P* value is plotted in each graph). MSC indicates mesenchymal stem cells; MI, myocardial infarction; EDV, end‐diastolic volume; ESV, end‐systolic volume; LV, left ventricle; ANOVA, analysis of variance.

**Figure 4.**
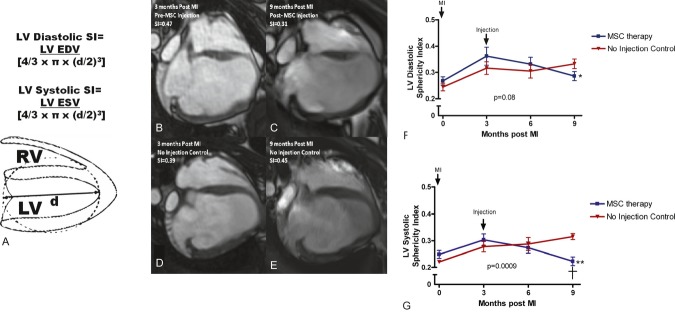
Allogeneic MSC therapy improves LV sphericity index. A, Formula for calculating the LV sphericity index (SI). B and C, CMR 4‐chamber diastolic SI images showing the LV chamber changing from the spherical shape of heart failure toward a more normal elliptical configuration after MSC therapy. D and E, No‐injection control animal's heart has worsening sphericiy (increased SI indicates a more spherical chamber, whereas a smaller SI models a more elliptical shape). Graphs of (F) diastolic SI and (G) systolic SI, showing improved sphericity after allogeneic MSC therapy. (*P=0.11, **P<0.01, †*P*<0.05 at 3 vs 9 months; between‐group ANOVA interaction *P* value is plotted in each graph). RV indicates right ventricle; LV, left ventricle; EDV, end‐diastolic volume; ESV, end‐systolic volume; MI, myocardial infarction; MSC, mesenchymal stem cells; CMR, cardiac magnetic resonance imaging; ANOVA, analysis of variance.

**Figure 5.**
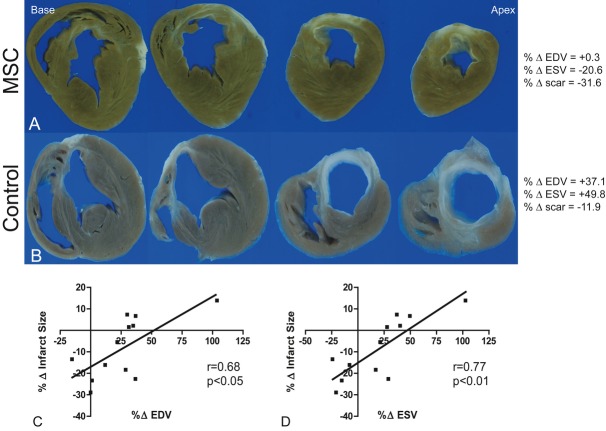
Infarct size reduction determines degree of left ventricle remodeling with allogenic MSC therapy. Example of (A) MSC‐treated heart showing a smaller LV chamber and scar (white tissue) compared with a (B) no‐injection control with a larger chamber and infarct. Correlation between the percentage change in infarct size from 3 months post‐MI (preinjection) to 9 months post‐MI (6 months postinjection) with the percentage change in (C) EDV and (D) ESV suggests that infarct size reduction with MSC therapy drives reverse remodeling in ischemic cardiomyopathy. MSC indicates mesenchymal stem cells; LV, left ventricle; MI, myocardial infarction; EDV, end‐diastolic volume; ESV, end‐systolic volume.

Ventricular remodeling after MI manifests by increased ventricular volumes (EDV and ESV) and geometrical alteration of the LV cavity from an elliptical to a spherical shape. Both placebo and MSC‐treated animals showed significant increases in ESV and EDV during the first 3 months after MI (Figure 3C and 3D), consistent with previous studies of remodeling after MI.^3^ MSC‐treated animals showed no increase in EDV (*P*=0.08) or ESV (*P*=0.28) from preinjection (3 months post‐MI) to 6 months postinjection, whereas controls had progressive dilatation in both EDV (*P*=0.0002) and ESV (*P*=0.0002) (Figure 3C and 3D). Sphericity index (SI), a metric that models the geometric configuration of the LV, showed MSC‐treated hearts were restored toward a more normal elliptical shape, whereas control animals' LV geometry remained spherical (Figure 4). MSC‐treated animals had improved systolic SI (*P*=0.003) and a trend toward improved diastolic SI (*P*=0.11), whereas no‐injection controls' systolic SI (*P*=0.15) and diastolic SI (*P*=0.56) were unchanged. Furthermore, correlation in percentage change in infarct size with EDV (*r*=0.68; *P*=0.01) and ESV (*r*=0.77; *P*=0.001) suggests that scar size reduction with MSC therapy regulates ventricular remodeling (Figure 5).

**Figure 6.**
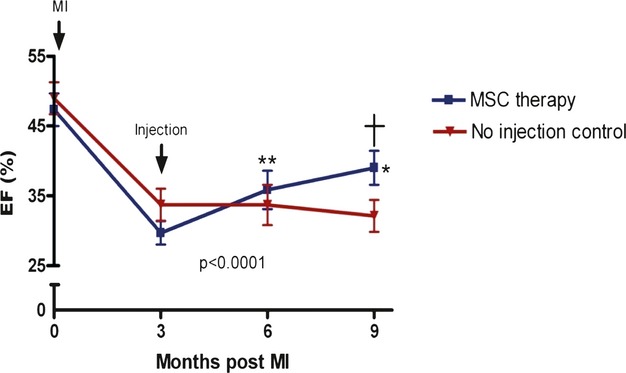
Progressive improvement in LV function with allogeneic MSC therapy. A, MSC therapy showed improved LVEF at 3 months postinjection, which progressively increased at 6 months postinjection. (*P<0.0001, **P<0.01 at 3 vs 6 months, †*P*<0.05 at 6 vs 9 months; between‐group ANOVA interaction *P* value plotted in the graph). LV indicates left ventricle; MSC, mesenchymal stem cells; LVEF, left ventricular ejection fraction; MI, myocardial infarction; EF, ejection fraction.

Accompanying these favorable effects on LV structure were substantial improvements in EF (Figure 6). Ejection fraction increased from 29.69±1.68% to 35.85±2.74% 3 months post‐MSC injection and progressed to 39.02±2.42% 6 months post‐MSC injection (*P*=0.0001), whereas controls had persistently depressed EF during follow‐up from 3 to 6 months post‐MI (*P*=0.33).

## Discussion

Here, we report the long‐term results of allogeneic MSC therapy in a swine model of ischemic cardiomyopathy. The effects of MSCs on scar size reduction are durable and progressive, support reverse remodeling, and are associated with improvements in LV function. In addition, we present an imaging platform that integrates 3‐dimensional CMR reconstruction of the LV with real‐time EAM viability data to guide transendocardial injection of MSCs to scarred areas of myocardium.

MSCs have been studied for decades and have an excellent safety profile.^10^ Taking advantage of the unique immunomodulatory properties of MSCs, allogeneic transplantation is well tolerated.^11,19^ Previous large animal studies have shown scar size reduction can manifest as early as 3 days after MSC injection, with the longest follow‐up being 90 days.^11,20^ Here, we studied swine for 6 months after MSC therapy in a large animal model of chronic ischemic cardiomyopathy and showed a durable 25% reduction in infarct size with MSC therapy (Figure 2). Accompanying this reduction in scar size in MSC‐treated animals was improved LV chamber volumes and geometric configuration toward a more normal elliptical shape. Consistent with the natural course of a large myocardial infarction, control animals continued to undergo progressive dilatation and the LV chamber progressively became more spherical in shape. Indeed, infarct size reduction after MSC therapy appears to play an important role in the favorable effects on ventricular remodeling (Figure 5). Heart failure is a progressive disease with a very poor prognosis, and MSC therapy may be capable of significantly altering adverse ventricular remodeling in the failing heart.

These findings offer additional insights into the findings of the POSEIDON trial,^12^ a comparison of allogeneic and autologous MSCs in patients with ischemic cardiomyopathy. Indeed, those patients experienced reductions in infarct size and improvements in the sphericity index. Determining the time course of the reduction and the sustainability in humans will help to guide the implementation of this therapeutic strategy.

The molecular and cellular mechanisms for scar size reduction with MSC therapy remain controversial. Engraftment and differentiation into new cardiomyocytes has been reported,^11,21^ but this is a rare event and cannot completely account for the substantial amount of scar size reduction seen. MSCs are capable of secreting numerous growth factors along with cytokines that may play a paracrine role. MSCs produce matrix metalloproteinases, enzymes with proteolytic activity that may physically remove scar tissue and allow regeneration of new cardiomyocytes.^22–23^ MSCs have long been known to interact and cooperate with other cells; however, their ability to stimulate endogenous cardiac stem cells to proliferate and differentiate is emerging as an exciting new mechanism of action.^20^ We recently reported that combining bone marrow–derived MSCs with c‐kit+ cardiac stem cells substantially improves scar size reduction compared with either cell type administered alone.^24–25^ Furthermore, it may be possible that MSCs stimulate adult cardiomyocytes to reenter the cell cycle.^20^ Indeed, the mechanism of action of MSCs is likely a result of several physiological mechanisms working together to repair damaged myocardium.

An important aspect of stem cell therapy for the heart is targeted delivery of cells to scarred regions of myocardium. Intramyocardial injection has been shown to produce the highest retention of cells,^26^ and EAM uses local voltage to guide an intramyocardial injection catheter to scarred myocardium, where stem cells are needed most. Strategies to integrate multimodality imaging can improve targeted delivery, and here we merged CMR angiography–derived 3‐dimensional anatomical reconstruction with EAM unipolar voltage to guide intramyocardial injection of MSCs. Integrating CMR and EAM showed that LV reconstruction volumes vary by imaging technique, highlighting the importance of using multimodality imaging for targeted cell delivery. As such, advanced imaging techniques will continue to play an important role for detailed cardiac phenotyping in stem cell therapy for heart failure.

The Gottingen swine model used here has emerged as highly representative of human ischemic cardiomyopathy^3,11^ with the exception that in this model, and as shown here, actual reductions in LV volumes are less evident than infarct size reductions. We attribute this difference to the fact that although we are using adult mini‐swine, they still experience some growth, thus the reversal of LV remodeling manifests as a prevention of ongoing increases in LV volumes as opposed to a reduction, per se. Despite this limitation, the findings here are likely to have implications for humans with ischemic cardiomyopathy.

The dosage of MSC therapy for this large animal study was chosen based on previous work by our group in a similar large animal model that showed that 200 000 autologous MSCs had greater effects on scar size than lower doses.^27^ Interestingly, our recent POSEIDON clinical trial studying allogeneic MSCs demonstrated that lower doses appear to be more beneficial than higher doses.^12^ The differences in response to cell dosage between these 2 studies could be related to factors specific to species (swine versus humans) or due to cell sources (allogeneic versus autologously derived MSCs). Indeed, further work is required to determine optimal MSC dosage.

In conclusion, MSCs reverse ventricular remodeling through durable infarct size reduction in swine with ischemic cardiomyopathy. These favorable changes in LV structure with MSC therapy are accompanied by long‐term improvements in cardiac function. Together these findings support the ongoing biological activity of cell therapy to create reverse remodeling in a process driven by infarct size reduction that occurs progressively. As such, infarct size and measures of remodeling are optimal end points for clinical trials.
